# Endoscopic Hematoma Evacuation Under Local Anesthesia for Acute Intracerebral Hemorrhage in Older Patients: A Case Series

**DOI:** 10.7759/cureus.80307

**Published:** 2025-03-09

**Authors:** Takahiro Tsuchiya, Tsukasa Koike, Atsumi Takenobu, Akio Morita, Akira Teraoka

**Affiliations:** 1 Department of Neurosurgery, Teraoka Memorial Hospital, Fukuyama, JPN; 2 Department of Neurosurgery, Tokyo Rosai Hospital, Ota-ku, JPN

**Keywords:** elder, endoscopic hematoma evacuation, intracerebral hemorrhage, local anesthesia, older patients

## Abstract

Intracerebral hemorrhage (ICH) is a severe neurological condition with a poor prognosis, and surgical intervention is often necessary in cases with significant hematoma volume. With the progression of a super-aging society, the incidence of ICH in older patients is increasing. However, traditional craniotomy under general anesthesia poses a high risk to older patients, thereby limiting its use. Recently, minimally invasive techniques have become widely used. Endoscopic hematoma evacuation can be performed under local anesthesia, which may minimize invasiveness and reduce the risk of complications associated with general anesthesia in older patients. However, only a few studies have specifically addressed endoscopic hematoma evacuation under local anesthesia for acute ICH in older patients, and the optimal protocol for surgical procedures and perioperative management remains unclear. The aim of this study is to highlight the technical considerations and potential benefits of endoscopic surgery for ICH in older patients. This study presents a case series of five patients aged ≥ 70 years who underwent endoscopic hematoma evacuation under local anesthesia for acute ICH. The median hematoma removal rate was 95.8%, with no postoperative re-bleeding or mortality. 3D visualization technology using preoperative computed tomography images not only facilitates the initiation of surgery but also assists with intraoperative support for accurate hematoma localization. In older patients, preoperative insertion of a nasogastric tube and the use of a transparent drape were effective in ensuring intraoperative airway management. Our findings suggest that endoscopic hematoma evacuation under local anesthesia is a safe and effective treatment option for older patients, offering reduced surgical invasiveness while maintaining high removal rates.

## Introduction

Intracerebral hemorrhage (ICH) is a devastating condition with a poor prognosis, and surgical hematoma evacuation remains the primary treatment for patients with significant hematoma volume and symptomatic presentation [1,2]. With the progression of a super-aging society, the incidence of ICH in older patients is increasing [3]. However, in older patients, traditional craniotomy under general anesthesia poses a high risk of complications and has limited applicability [4]. In recent years, there has been a shift toward less invasive techniques such as endoscopic and stereotactic procedures, which have gained popularity [5-8]. Among these, endoscopic hematoma evacuation can be performed under local anesthesia, potentially reducing the complications associated with general anesthesia in older patients. Previous studies have reported that endoscopic hematoma evacuation can shorten operation time, minimize invasiveness, maintain complication rates comparable to those of traditional methods, and potentially improve outcomes [9-11]. However, there is a paucity of literature specifically addressing endoscopic hematoma evacuation under local anesthesia for acute ICH in older patients [9,12]. Consequently, optimal protocols for surgical procedures and perioperative management remain unclear. This study aimed to present a case series from our institution and highlight the technical considerations, perioperative management, and potential benefits of endoscopic surgery for ICH in older patients.

## Case presentation

Case series

Patients aged ≥ 70 years who underwent endoscopic hematoma evacuation under local anesthesia for acute ICH at Teraoka Memorial Hospital between January 2022 and December 2024 were retrospectively reviewed. The inclusion criteria for this study were as follows: patients with ICH whose hematoma volume was greater than 30 ml and who exhibited a mass effect or hydrocephalus. Patients with ICH resulting from trauma, vascular malformations, ruptured aneurysms, tumor bleeding, impaired coagulation, vasculitis, or hemorrhagic transformation due to ischemic stroke were excluded. This study was approved by the Ethics Committee of Teraoka Memorial Hospital (FY2024-16), which waived the requirement for informed consent. The study followed the opt-out method for obtaining consent, as detailed on the website of Teraoka Memorial Hospital.

**Figure 1:**
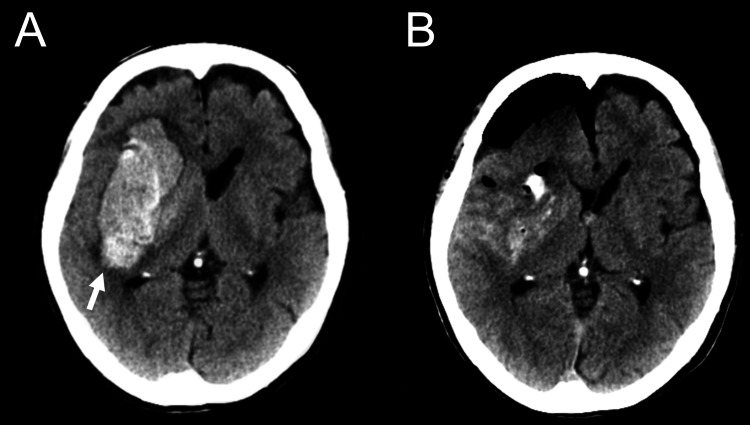
Preoperative and postoperative CT images of case 1. A 70-year-old female patient was admitted to our institution with left paralysis and loss of consciousness. CT imaging revealed an intracerebral hemorrhage of 89.0 ml in the right putamen (A). Subsequently, endoscopic hematoma evacuation was performed under local anesthesia. Postoperative CT imaging revealed that 95.8% of the hematoma had been successfully removed (B).

Five consecutive patients underwent endoscopic hematoma evacuation under local anesthesia for acute ICH. During this period, four patients underwent endoscopic hematoma evacuation under general anesthesia. These patients were considered to have a high risk of airway obstruction due to severe disturbance of consciousness, so the surgery was performed under general anesthesia. In cases where substantial cerebral edema was present and decompression surgery was required, traditional craniotomy was performed under general anesthesia. The clinical characteristics and outcomes of the five patients who underwent endoscopic hematoma evacuation under local anesthesia for acute ICH are shown in Table 1. The median age of the patients was 79 years (range: 70-91 years); one patient (20%) was female; and the location of the hematoma was the putamen in two cases (40%), subcortical in two cases (40%), and cerebellar in one case (20%). Representative preoperative and postoperative computed tomography (CT) images of case 1 are shown in Figure 1. The median preoperative Glasgow Coma Scale (GCS) score was 11 (range: 10-13). The hematoma volume before and after surgery was volumetrically measured using 3D Slicer version 5.8.0 software. The median hematoma volume before surgery was 84.1 ml (range: 41.9-111.8 ml), and the median hematoma removal rate was 95.8% (range: 82.4-97.4%). No postoperative re-bleeding or complications related to the surgical procedure were observed in any patient. The median modified Rankin scale (mRS) score at discharge was 4 (range: 4-5), and all patients were alive at discharge. All patients were followed up with a median follow-up period of five months.

**Table 1:** Clinical characteristics and outcomes of five patients who underwent endoscopic hematoma evacuation under local anesthesia for acute intracerebral hemorrhage. F: female; M: male; R: right; L: left; GCS: Glasgow Coma Scale; mRS: modified Rankin scale.

	Year	Sex	Side	Location	Preoperative GCS score	Hematoma volume (ml)	Hematoma removal rate (%)	Operation time (minutes)	Postoperative re-bleeding	mRS score at discharge	Follow-up period (months)
Case 1	70	F	R	Putamen	11	89.0	95.8	97	No	4	7
Case 2	91	M	L	Subcortex	11	111.8	97.4	96	No	4	6
Case 3	88	M	L	Subcortex	10	84.1	92.3	73	No	5	5
Case 4	79	M	R	Putamen	13	55.5	82.4	80	No	4	4
Case 5	77	M	R	Cerebellum	10	41.9	96.3	94	No	5	4

Preoperative management

Upon admission, nicardipine was administered to maintain the systolic blood pressure within the normal range, specifically below 140 mmHg. This blood pressure management protocol was initiated at the time of admission to our institution and was implemented throughout the surgical procedure.

**Figure 2:**
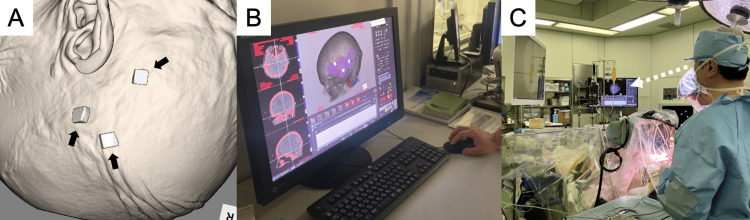
The utilization of 3D images for preoperative planning and intraoperative reference. The attachment of three or four CT-visible markers to the patient's head skin facilitates accurate localization of the hematoma. We referred to this technique as "Marking CT" (A). The 3D images are created using Ziostation2 software (Ziosoft Inc., Tokyo, Japan), performed by trained radiographers (B). Surgeons utilize these 3D images for intraoperative reference and to maintain orientation during endoscopic hematoma evacuation (C).

Endoscopic hematoma evacuation requires careful consideration of the craniotomy location. Although the shallowest point of a hematoma is typically the first choice, it is crucial to ensure that the entire hematoma is evacuated from the chosen location. As head pins are not used for head fixation under local anesthesia, navigation systems are challenging to use. To address this challenge, preoperative CT was performed with markers. The attachment of three or four CT-visible markers to the skin of the patient’s head facilitates accurate localization of the hematoma, obviating the need for navigation systems. We referred to this technique as "Marking CT" (Figure 2A). This CT scan was used to generate 3D visualizations before surgery, aiding intraoperative orientation. The 3D images were created using Ziostation2 software (Ziosoft Inc., Tokyo, Japan), a task initially performed by doctors but now delegated to trained radiographers (Figure 2B). This task-shifting allows radiographers to complete 3D imaging in 15-20 minutes while surgeons prepare for the surgery, potentially reducing the time to surgery in emergency cases such as ICH ones. Surgeons utilize these 3D images for both preoperative planning and intraoperative reference, finding them particularly useful for determining craniotomy locations and maintaining orientation during endoscopic hematoma evacuation procedures (Figure 2C). Moreover, it is imperative to employ CT angiography to examine for vascular anomaly and underlying diseases of ICH, such as aneurysms, prior to surgery.

Airway management is critical in procedures performed under local anesthesia, especially in older patients. The insertion of a nasogastric tube prior to entering the operating room to remove gastric contents reduces the risk of aspiration by vomiting. A nasal airway is also useful for preventing airway obstruction due to tongue root subsidence. Appropriate sedation and analgesia were vital, and intravenous diazepam (5-10 mg) and pentazocine (7.5-15 mg) were used for sedation. The use of adequate local anesthesia is paramount to ensure effective pain management. A transparent drape was employed to facilitate the observation of patient movement and respiration, and strict vital sign monitoring was maintained throughout the procedure.

Operative procedure

**Figure 3:**
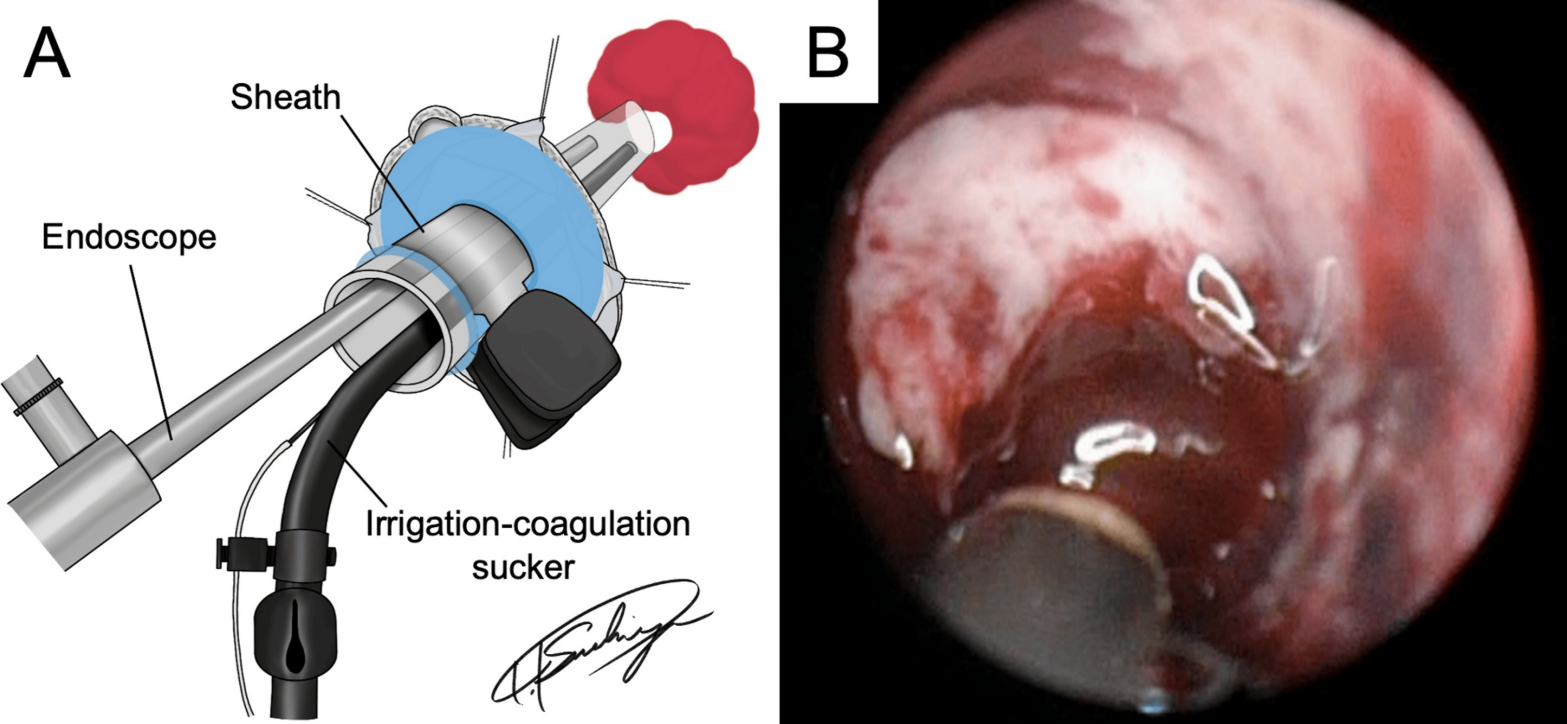
Intraoperative findings of endoscopic hematoma evacuation under local anesthesia for acute intracerebral hemorrhage. Illustration shows the procedure of endoscopic hematoma evacuation using a transparent sheath (Neuroport; Olympus Medical Systems Corp., Tokyo, Japan), an endoscope (2.7 mm, 0° angle; Olympus Medical Systems Corp.), and an irrigation-coagulation sucker (4.0 mm; Fujita Medical Instrument, Tokyo, Japan) (A; Image credits: Takahiro Tsuchiya). The sheath is rotated and the hematoma that enters the sheath is aspirated with an irrigation-coagulation sucker (B).

A linear or curved skin incision was made based on preoperative marking CT. A small craniotomy measuring approximately 3-4 cm was performed using two burr holes, followed by a dural incision. Ultrasound imaging was used to confirm the optimal location for a corticotomy to access the hematoma. Following corticotomy, a transparent sheath (Neuroport, Olympus Medical Systems Corp., Tokyo, Japan) was inserted along with an endoscope (2.7 mm, 0° angle; Olympus Medical Systems Corp.). Once hematoma outflow was confirmed, an assistant secured the sheath in place (Figure 3A). The sheath was rotated to facilitate natural hematoma drainage and then aspirated without aggressive pursuit to avoid deep bleeding, which was difficult to control endoscopically (Figure 3B). Hemostasis was achieved using an irrigation-coagulation sucker (4.0 mm; Fujita Medical Instrument, Tokyo, Japan) capable of simultaneous irrigation and monopolar coagulation. Hematoma evacuation was guided by the prepared 3D images to maintain spatial orientation. Upon completion, adequate decompression and hemostasis were verified before dural closure, bone flap fixation using titanium plates, and skin suturing. During the closure period, administration of additional intravenous diazepam or pentazocine may be considered in patients experiencing pain or inadequate sedation. As previously mentioned, blood pressure control with nicardipine is important for preventing postoperative re-bleeding. In addition to re-bleeding, it is vital to manage postoperative patients meticulously to prevent postoperative pneumocephalus or worsening of cerebral edema.

## Discussion

Endoscopic surgery for ICH offers significant advantages over conventional craniotomy owing to its minimally invasive nature [13]. Studies comparing endoscopic surgery and craniotomy under general anesthesia have demonstrated that endoscopic surgery is safe and effective, resulting in shorter operation times, reduced blood loss, and decreased intensive care unit and hospital stays [8,14,15]. Although the number of studies reporting the outcomes of endoscopic hematoma evacuation under local anesthesia is limited, it has been suggested that this approach may further reduce operation times compared with craniotomy [9]. Hayashi et al. reported a series of 28 patients who underwent endoscopic hematoma evacuation under local anesthesia; 11 (39.3%) patients had a hematoma removal rate of less than 60%, with lower rates observed by less experienced surgeons and patients with end-stage chronic renal failure or liver cirrhosis [12]. Our case series achieved a median removal rate of 95.8%, which is notably higher than that reported previously. One reason for this is probably that the intraoperative imaging support provided by the 3D images helped the surgeon to accurately ascertain the location of the hematoma. The only case in our series with less than a 90% removal rate was case 4, with a putaminal hemorrhage extending into the thalamus, which was intentionally left unevacuated due to its deep location. Regarding the outcomes, our case series exhibited a median mRS score at discharge of 4, consistent with previous reports, reflecting the generally poor neurological prognosis of ICH patients [9,12]. While previous studies have reported mortality rates of 17.9-20.0%, our case series had no mortality, likely due to the inclusion of less severe cases with a median preoperative GCS score of 11 (range: 10-13) [11,12].

Local anesthesia is an effective approach to reduce the invasiveness of surgical procedures in older patients. While there are no specific studies on endoscopic hematoma evacuation under local anesthesia for acute ICH in older patients, endoscopic surgery under local anesthesia for acute subdural hematoma in older patients (aged ≥ 70 years) has been reported to be safe and effective [16,17]. Performing surgery under local anesthesia not only minimizes patient invasiveness but also allows for early initiation of rehabilitation, potentially leading to improved neurological function and reduced hospital stay [8]. Airway management is crucial in local anesthesia surgeries with preoperative nasogastric tube insertion, and transparent drapes are useful for ensuring airway access and management.

ICH causes severe neurological impairment at the time of onset due to hematoma formation, making complete neurological recovery unlikely, even with complete hematoma evacuation [18]. Surgical intervention is primarily aimed at saving lives rather than significant neurological improvement. In older patients, careful consideration of treatment indications is necessary because of their poor general condition and multiple comorbidities. However, treatment decisions should not be based solely on age; it is crucial to identify patients who may benefit from surgical intervention and carefully consider treatment options [19]. This concept is supported by evidence showing that a significant proportion of ICH patients aged ≥ 80 years can have favorable outcomes, with 35% being discharged to home or rehabilitation facilities [20].

This study had several limitations that warrant consideration. As this was a single-arm retrospective analysis, it lacked comparisons with other treatment options, such as craniotomy or conservative treatment. Additionally, it was not possible to make comparisons between the selection criteria for this approach and those for conventional treatment. Finally, the small sample size limits the extent to which the treatment outcomes can be comprehensively discussed. Further accumulation of clinical experience and follow-up data, and also further research to validate these findings in larger, comparative studies will help to elucidate the efficacy and significance of this treatment modality in older patients.

## Conclusions

The number of ICH cases in older patients is increasing, and minimally invasive treatment is desirable in this population because of the high risk of complications. Endoscopic hematoma evacuation under local anesthesia can be performed safely and effectively in older patients with acute ICH. Strict management of the airway can ensure safe treatment, and the use of 3D images for intraoperative orientation can improve the hematoma removal rate.

## References

[1] Gregson BA, Broderick JP, Auer LM (2012). Individual patient data subgroup meta-analysis of surgery for spontaneous supratentorial intracerebral hemorrhage. Stroke.

[2] Pradilla G, Ratcliff JJ, Hall AJ (2024). Trial of early minimally invasive removal of intracerebral hemorrhage. N Engl J Med.

[3] van Asch CJ, Luitse MJ, Rinkel GJ, van der Tweel I, Algra A, Klijn CJ (2010). Incidence, case fatality, and functional outcome of intracerebral haemorrhage over time, according to age, sex, and ethnic origin: a systematic review and meta-analysis. Lancet Neurol.

[4] Soleman J, Ullmann M, Greuter L, Ebel F, Guzman R (2021). Mortality and outcome in elderly patients undergoing emergent or elective cranial surgery. World Neurosurg.

[5] Nakano T, Ohkuma H, Ebina K, Suzuki S (2003). Neuroendoscopic surgery for intracerebral haemorrhage--comparison with traditional therapies. Minim Invasive Neurosurg.

[6] Cho DY, Chen CC, Chang CS, Lee WY, Tso M (2006). Endoscopic surgery for spontaneous basal ganglia hemorrhage: comparing endoscopic surgery, stereotactic aspiration, and craniotomy in noncomatose patients. Surg Neurol.

[7] Montes JM, Wong JH, Fayad PB, Awad IA (2000). Stereotactic computed tomographic-guided aspiration and thrombolysis of intracerebral hematoma : protocol and preliminary experience. Stroke.

[8] Haseeb A, Shafique MA, Mustafa MS (2024). Neuroendoscopic versus craniotomy approach in supratentorial hypertensive intracerebral hemorrhage: an updated meta-analysis. World Neurosurg.

[9] Katsuki M, Kakizawa Y, Nishikawa A, Yamamoto Y, Uchiyama T (2020). Endoscopic hematoma removal of supratentorial intracerebral hemorrhage under local anesthesia reduces operative time compared to craniotomy. Sci Rep.

[10] Kellner CP, Song R, Pan J (2020). Long-term functional outcome following minimally invasive endoscopic intracerebral hemorrhage evacuation. J Neurointerv Surg.

[11] Flores J (2021). Evacuation of intracerebral hematomas by neuroendoscopy: results in a series of cases. Peruv J Neurosurg.

[12] Hayashi T, Karibe H, Akamatsu Y (2019). Endoscopic hematoma evacuation for intracerebral hemorrhage under local anesthesia: factors that affect the hematoma removal rate. World Neurosurg.

[13] Zhao YN, Chen XL (2016). Endoscopic treatment of hypertensive intracerebral hemorrhage: a technical review. Chronic Dis Transl Med.

[14] Noiphithak R, Yindeedej V, Ratanavinitkul W, Duangprasert G, Nimmannitya P, Yodwisithsak P (2023). Treatment outcomes between endoscopic surgery and conventional craniotomy for spontaneous supratentorial intracerebral hemorrhage: a randomized controlled trial. Neurosurg Rev.

[15] Zhan Y, Zou X, Wu J (2023). Neuroendoscopy surgery for hypertensive intracerebral hemorrhage with concurrent brain herniation: a retrospective study of comparison with craniotomy. Front Neurol.

[16] Yokosuka K, Uno M, Matsumura K (2015). Endoscopic hematoma evacuation for acute and subacute subdural hematoma in elderly patients. J Neurosurg.

[17] Katsuki M, Kakizawa Y, Nishikawa A, Kunitoki K, Yamamoto Y, Wada N, Uchiyama T (2020). Fifteen cases of endoscopic treatment of acute subdural hematoma with small craniotomy under local anesthesia: endoscopic hematoma removal reduces the intraoperative bleeding amount and the operative time compared with craniotomy in patients aged 70 or older. Neurol Med Chir (Tokyo).

[18] Mendelow AD, Gregson BA, Fernandes HM (2005). Early surgery versus initial conservative treatment in patients with spontaneous supratentorial intracerebral haematomas in the International Surgical Trial in Intracerebral Haemorrhage (STICH): a randomised trial. Lancet.

[19] Fujita K, Tanaka K, Yamagami H (2021). Outcomes of large vessel occlusion stroke in patients aged ≥90 years. Stroke.

[20] Forman R, Slota K, Ahmad F, Garg R, John S, Da Silva I, Koffman L (2020). Intracerebral hemorrhage outcomes in the very elderly. J Stroke Cerebrovasc Dis.

